# Factors determining dry deposition of total mercury and organic carbon in house dust of residents of the Tri-city and the surrounding area (Baltic Sea coast)

**DOI:** 10.1007/s11869-017-0471-2

**Published:** 2017-04-01

**Authors:** Kinga Wiśniewska, Anita Urszula Lewandowska, Agnieszka Witkowska

**Affiliations:** Institute of Oceanography, University of Gdansk, Al. Marszałka J. Piłsudskiego 46, 81-378 Gdynia, Poland

**Keywords:** Mercury, Carbon, Deposition, Household dust, Tri-city, Poland

## Abstract

The purpose of this study was to find out what factors determine the deposition levels of mercury and organic carbon in household dust in the Tri-city region (southern Baltic Sea coast). Analyses were performed on samples collected over the period of 2 years, from 2013 to 2015, always in the heating season. The deposition of organic carbon was between 4and 210 mg m^−2^ month^−1^, while mercury deposition ranged from 4 to 1336 ng m^−2^ month^−1^. Deposition of mercury in household dust during the heating season was three times lower and deposition of organic carbon one and a half times lower than outdoor deposition obtained in the Baltic Sea region by other researchers. In the non-heating period, deposition of mercury in household dust was similar to outdoor deposition while deposition of OC was one and a half times higher. Both of the analyzed dust components reached higher deposition in rural areas than in cities, and both mercury and organic carbon were found to have higher deposition in single-family houses than in buildings housing several families. The increased level of OC was conditioned by the vicinity of the building to a road or street with a high level of traffic, and dust collected on the ground floor had higher Hg depositions. The presence of plants and pets, as well as smoking more than ten cigarettes per day, resulted in higher depositions of both the compounds present in household dust within the Tri-city region.

## Introduction

Outdoor atmosphere is often monitored and it could be perceived as the sole source of inhaled toxins; nevertheless, people spend more time in enclosed spaces, both at work and at home. An average woman between the ages of 25 and 60 stays indoors 84% of the time, while for a man, it is 59%. An additional 4–7% of each day is spent inside various means of transportation (Schweizer et al. 2007; Semple et al. 2012; Buoanno et al. 2014). This statistic affects city dwellers more than the rural population (38 and 34% respectively) (www.stat.gov.pl) and can be attributed to urbanization, lifestyle, and diet of the population (Jie et al. 2013). This has a significant impact on people’s health and quality of life and may be the reason for an increase in noted diseases of both young and middle-aged people. Observations conducted over several years show that especially women spending a lot of time at home have more health problems. It is because in the long term, remaining in an indoor environment at home and at work is conducive to increased exposure to toxic and harmful substances (Du et al. 2015). In an indoor atmosphere these substances become deposited on different surfaces along with other components such as mold spores, mites, and their excrement or discharge. These substances can be introduced into the human body by normal daily tasks using items on which they have been deposited on, or absorbed by (including but not limited to eating at tables or children playing with toys). Harmful and toxic substances can return to the air due to resuspension and reach the respiratory system directly. Particles released from some indoor sources show high total lung deposition fraction (Vu et al. 2016).

Dust contains significant information about the substances that people absorb. In household dust, there can be found 80 different organic compounds (including PAHs, PCB) as well as metals (e.g. cadmium, lead, etc.) (Hogervorst et al. 2007; Knobeloch et al. 2012). Therefore, toxic and hazardous substances presented in household dust are one of the most influential factors that affect indoor air quality. Mercury and organic carbon compounds are two particularly dangerous substances penetrating into the human body through inhaled air at home and at work. Not only is mercury a metal for which there is no physiological need in the human organism, it is actually toxic to humans in all forms. Mercury compounds have a negative influence on the nervous and immunological systems (Tucaliuc et al. 2014), as well as the potential to affect the respiratory system, kidneys, skin, and the eyes (Lu et al. 2009). At home and at work, mercury can be present as a result of the use of fluorescent lights and, until recently, mercury thermometers (Carpi and Chen 2001). As of 2009, Poland no longer produces mercury thermometers (www.gios.gov.pl). Another way that mercury is emitted into the atmosphere is during the production of power and cement (Zheng et al. 2012). Carbon compounds in the atmosphere come mainly from the burning of various fuels (biomass, fossil fuels, unleaded petrol), but they are also components of repellents used in upholstered furniture (http://www.cohiba-project.net). Many organic carbon compounds are known to have carcinogenic and mutagenic properties (Adar and Kaufman 2007).

There are numerous potential sources of indoor air pollutants, including for example, the heating system used in buildings, its location in relation to thoroughfares or industrial centers, and the smoking of tobacco (Schweizer et al. 2007; Semple et al. 2012). In addition to the aforementioned factors, the quality of the surrounding air can also be affected by the use of chemical cleaning products, the frequency of cooking, and the type of food being prepared (Carpi and Chen 2001). Therefore, the composition of household dust can depend on the room’s application and place of deposition. The dust found on the floor has a different composition in comparison to dust located at elevated places like windowsills or rack’s due to cleaning frequency. The knowledge on factors influencing the presence and high concentrations of organic carbon or mercury deposited in dust is infinitesimal. Thus, we were interested to comprehend and quantify exposure on these substances deposited in household dust of residents in the Tri-city and surrounding areas located in the Polish coastal zone of the southern Baltic Sea. The main goal of this study is to determine which factors influence the deposition levels of mercury and organic carbon in household dust. The studies were carried out on a group of a few dozen university students, who burdened by studies, often taking additional jobs, using stimulants, and living in places of varied standards formed an interesting study group.

## Materials and methods

### Characterization of the study area

**Fig. 1:**
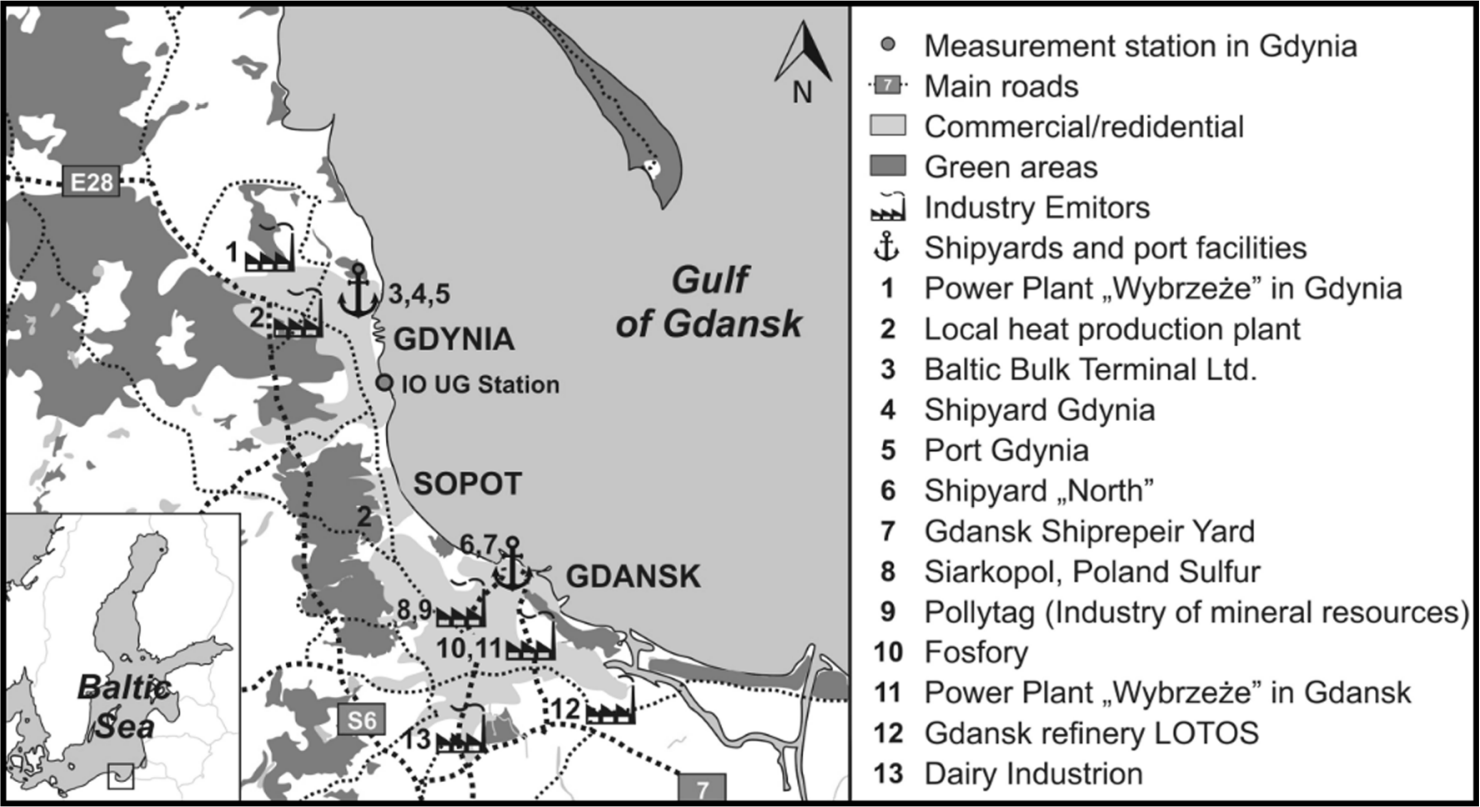
A map showing potential sources of carbon and mercury in the atmosphere of the Tri-city region

The dust samples were collected in the years 2013–2015 on a monthly basis, each time during the heating season, in the home environment (*N* = 62 samples for Hg and *N* = 41 samples for OC) and at work (*N* = 7 for both Hg and OC). The Tri-city agglomeration is located in the north of Poland, in the coastal zone of the Southern Baltic, on the Gulf of Gdansk (Fig. 1).

The most important parameters determining the distribution of pollutants in the seaside atmosphere of the Southern Baltic are the speed and direction of wind, air temperature, and humidity. (Lewandowska et al. 2013). The study area is a meeting place for marine polar and continental polar air masses, and, depending on the season, they cause either the warming or the cooling of the climate. The winters here can be relatively warm, while the summers tend to be cold with small temperature amplitudes (20–25 °C) and high humidity (60–75% on average) (Czernecki and Miętus 2015). The average wind speed of 1.5–2.9 ms^−1^ is responsible for bringing in pollutants of both local and regional character (Lewandowska et al. 2010; Lewandowska and Falkowska 2013).

The best-developed sectors of industry are the shipbuilding, food production, and electrical industries (Fig. 1). This area is also home to Siarkopol S.A., a company managing the exportation of sulfur fossil and providing transshipment and storage of liquid products: petrochemical, chemical, and vegetal; Pollytag S.A. which produces construction aggregate made of fly ashes; Fosfory which is a producer of mineral fertilizers and chemical products, widely used in agriculture as well as fruit and vegetable growing; and LOTOS S.A. which operates in the extraction, refinery, and sale of petroleum products. This latter produces and provides unleaded petrol, diesel fuel, light heating oil, aircraft fuel, and heavy heating oil and also specialize in the production and sale of lubricating oils and asphalt. Together with the aforementioned plants, the community sector is also of great significance for the quality of the surrounding air. Heating in this sector involves the use of coal (53%) and lignite (35%) (www.infoekopomorskie.pl). Given that the Tri-city agglomeration is located in the coastal zone of the Baltic, land and sea transportation also play an important role in air pollution.

### Sample collection

Samples were collected by 69 students in home and work environments during the heating season (February and March) from 2013 to 2015. A passive method of home dust collection was used involving quartz Whatman QM-A filters of 47 mm in diameter placed in Petri dishes. While the samples were being collected, two open dishes with filters were located in every house at a height of between 1.0 and 1.5 m above the floor. This was to simulate the level at which a sitting person would be breathing. The filters were located in places that were not directly exposed to potential sources of home pollutants.

Prior to use, the filters were roasted at 550 °C for a minimum of 5 h in order to remove mercury and organic compounds. Next, the filters were conditioned for 2 days (at 20 ± 2 °C and air humidity of 45 ± 5%) and weighed. After another day of conditioning, the filters were weighed again. The conditioning and weighing procedure was repeated after the collection of a dust sample. The difference in filter weight before and after collection indicated dust weight expressed in grams. All the weighing procedures were carried out on a RADWAG scale, of 10^−5^ g in accuracy. In each collection campaign, one blank sample was collected in order to eliminate contamination. The uncertainty of the weighing method for every pair of filters was <5.0% (at a certainty level of 99%).

Apart from collecting samples, students were asked to complete a questionnaire which included the following: number of household inhabitants, size of the apartment, type of building construction, distance from the street and level of traffic, type of heating used in the household, frequency of cooking and cleaning, presence of pets or plants, and number of cigarettes smoked. In addition, seven samples were obtained for the students’ work places: a carpentry workshop, an optical laboratory, a restaurant, a pub, a petrol station, a health clinic, and the oceanography science club room at the Oceanography Institute of the Gdansk University.

### Chemical analysis

#### Analyzing mercury in dust samples

The deposition of mercury in indoor dust was assayed using an AMA 254 mercury analyzer (Advanced Mercury Analyzer, Altec), using the method of atomic absorption spectrometry. The method does not require any special preparation of the samples (e.g., extraction, etching), which reduces the risk of them becoming contaminated (Száková et al. 2004). The precision and accuracy of the method were checked by analyzing certified reference material (NIST Standard Reference Material 2584; indoor dust, 5.20 μgHg g^−1^), and the obtained results for method repeatability were at a level of 96.8%. The detection limit, determined as standard deviation of the blank multiplied by three, was 0.005 ng g^−1^ (Bełdowska et al. 2012). To analyze mercury, a filter of 47 mm in diameter was used, out of which a piece measuring 1.5 cm^2^ had been cut and used for organic carbon analysis. The final mercury deposition, reduced by the value of the blank (0.020 ng m^−2^ month^−1^), was converted to dust weight and expressed in ng m^−2^ month^−1^. The uncertainty of the method for every pair of filters was <4.0% (at a certainty level of 99%).

#### Analyzing carbon in dust samples

Organic carbon in the dust samples collected indoors was analyzed using the thermo-optic method, using an OC-EC analyzer by Sunset Laboratory Inc., the EUSAAR2 protocol (European Supersites for Atmospheric Aerosol Research). The thermo-optic method enables a selective assay of organic carbon deposition with accuracy of up to 1 μg C (Cavalli et al. 2010). A rectangular piece of 1.5 cm^2^ was cut out of the quartz filter (47 mm in diameter) onto which the sample had been collected, placed in a quartz furnace, and then analyzed. All the results were decreased by the value of the blank, which did not exceed 3.0 μg per 1 cm^2^ of the filter. The final carbon deposition was converted to dust weight and expressed in mg∙g^−1^. The method’s limit of detection was established at 2 μg (*N* = 12) and analytical error was below 8% (for the confidence interval of 99%). The uncertainty of the method for every pair of filters was <5.0% (at a certainty level of 99%). Apart from automatic calibration (internal standard: 5.0% methane in a balance with analytically pure He), which takes place each time at the end of the second stage of the analysis, an external standard (analytically pure 99.9% sugar solution) was analyzed every 10–15 samples (Cavalli et al. 2010). The analytical error of the method was determined to be 4.5%. Additionally, an intercalibration was performed of the OC-EC method with the 13C isotope method (Université du Québec in Montreal), using a 2500 NC Elemental Analyzer. The obtained results were highly compatible, as confirmed by a high Pearson’s correlation coefficient (*r* > 0.9).

### Statistical treatment of the data

To verify the significance of the impact of the analyzed factors (e.g., distance from the street, level of traffic, type of heating used in the household, frequency of cooking and cleaning, presence of pets/plants, number of cigarettes smoked, etc.) on the mercury deposition in house dust, two tests were applied. The non-parametric U Mann-Whitney Test was applied to test differences between two sets of independent data and the Kruskal-Wallis test was used for more than two groups of independent variables. Analogous tests were applied for determining the influence of selected factors on the deposition of organic carbon in house dust. For all dependencies presented in the publication, the levels of tests’ significance have been considered to be important only when *p* value was less than 0.05. All the statistical analysis was performed using STATISTICA® Software (Version 12).

## Results and discussion

**Fig. 2:**
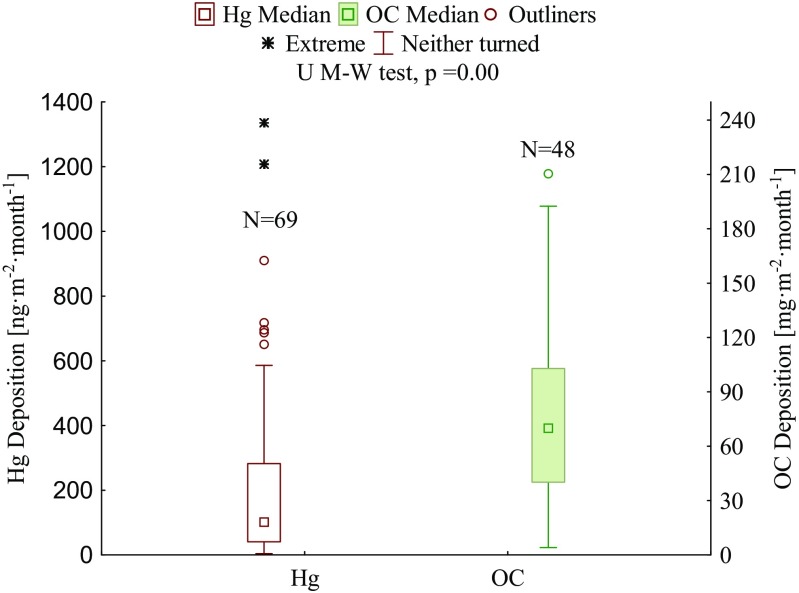
Mercury [ng m^−2^ month^−1^] and organic carbon [mg m^−2^ month^−1^] deposition in household dust

There have been more and more evidence of the adverse effects of air pollution in home environment, even at low levels of pollutants (Hulin et al. 2012). Still, the deposition of organic carbon and mercury in the indoor atmosphere is most often studied in terms of aerosols (e.g., Cao et al. 2005; Schweizer et al. 2007; Semple et al. 2012). Studies of these components in household dust have not been carried out as yet. In our research, the Mann Whitney U test showed statistically significant differences in mercury and organic carbon concentrations during measurement period (*p* = 0.0). The deposition of organic carbon (OC) in all dust samples (*N* = 48) collected over a period of 1 month ranged from 4 to 210 mg m^−2^ month^−1^ (median 70 mg m^−2^ month^−1^). Mercury deposition varied between 4 and 1336 ng m^−2^ month^−1^ (median 102 ng m^−2^ month^−1^, *N* = 69) (Fig. 2). Mercury deposition in samples collected at home and in workplaces were very similar (median 106 and 95 ng m^−2^ month^−1^, respectively). In the case of organic carbon, the median of deposition value was higher in household than in workplace dust (77 and 36 mg m^−2^ month^−1^, respectively). However, owing to the small number of samples representing the work environment and the lack of statistically significant differences between the two environments for both Hg and OC (*p* > 0.05, Mann-Whitney U test), they are described jointly in the following part of the present paper with the exception of outlying or extreme values, found in either of the two environments (home/work).

Similarly to the outdoor air, in house dust, mercury can be adsorbed onto carbon, a quality that enables it to reach the human body more easily, resulting in a higher potential health risk (Adar and Kaufman 2007). However, within the entire study period (between 2013 and 2015), no correlation was found between the analyzed dust components in samples collected in the Tri-city (*r* ^2^ = 0.01). Only in the samples collected in 2015 was statistically significant, directly proportional dependency between OC and Hg depositions (r^2^ = 0.80 *p* < 0.05) noted, and this suggested a common source of their origin during that measurement period. In 2015, the deposition of mercury was more than ten times lower than in the rest of the measurement period (22 and 288 ng m^−2^ month^−1^, respectively) while organic carbon deposition was of the same order of magnitude and just slightly lower (60 and 80 mg m^−2^ month^−1^). That year was not characterized by extremely low temperatures and did not stand out in terms of any other meteorological parameter which could suggest an intensified burning process for heating purposes. However, air pollution in a household is a mixture of particles and gases which are emitted both internally and externally, and those which were formed indoors as a result of reactions of gas precursors emitted both inside and outside the building (Morawska and Salthammer 2003; Meng et al. 2005). For this reason, the composition and toxicity of aerosols present in the atmosphere of enclosed areas are very complex and exhibit certain similarities to aerosols in the open air (Morawska et al. 2013). In order to establish what factors determined the deposition of mercury and organic carbon in indoor dust, and what situations lead to the binding of these two, both external and internal environmental factors were taken into account in the following part of this discussion.

## The influence of external environment on the deposition levels of mercury and organic carbon deposited in dust

As was considered by Ma and Harrad (2015), outdoor sources could emit more carcinogenic organic substances, compared to indoor sources. The infiltration of aerosols which are generated outdoor, into indoor air, can be estimated by the comparison of concentrations/depositions of indoor air and outdoor air (Mohammed et al. 2016). In this study, to compare indoor and outdoor deposition, the formula based on dry deposition was used (Rhode et al. 1980):

1 $$ {F}_{D RY}= C\cdot {V}_D $$

where *F*_*DRY*_—dry deposition flux of Hg or OC (in ng∙m^−2^∙month^−1^ and mg∙m^−2^∙month^−1^, respectively), C—Hg or OC concentration (in ng∙m^−3^ and mg∙m^−3^, respectively) (monthly averages of Hg and OC in aerosols), and *V*_*D*_—settling velocity (for aerosols between 1 and 10 μm in size 0.005 m s^−1^ and below 1 μm in size 0.0005 m s^−1^).

The median of mercury deposition in household dust was equal to 102 ng m^−2^ month^−1^. We were unable to find the literature on the deposition of mercury in house dust; hence, such a comparison could not be made. A previous study conducted in the center of Gdynia from 18.12.2007 to 15.12.2008 by Beldowska and coauthors (2012) enabled us to compare outdoor deposition of particulate mercury (in fine and coarse fraction) with present data of indoor mercury deposition. It was possible, given that in both cases, samples were analyzed using the same analytical method and that both, fine and coarse outdoor aerosols, were taken into account. We found, that outdoor and indoor depositions were of this same order of magnitude, regardless of the season outside. It might suggest a possibility of outdoor source contribution in indoor mercury deposition. However, the indoor deposition was three times lower than outdoor deposition obtained during the heating period (311 ng m^−2^ month^−1^) and just slightly lower than the during non-heating period (138 ng m^−2^ month^−1^). In air over the Tri-city agglomeration, the majority of particulate mercury (Hg (p)) originates from direct anthropogenic emissions, especially from heat and power production as well as gaseous mercury transformations in the atmosphere (Beldowska et al. 2012). This would explain why the deposition of mercury in the heating is twice as high as in the non-heating season, even more so as a majority of detached houses in the region are still heated by low-capacity domestic heating unit (DHU) (Beldowska et al. 2012). In addition, in the outdoor air, more dynamic changes of air chemical composition occurs due to changeable meteorological conditions (Lewandowska and Falkowska 2013; Vanos et al. 2015). In the coastal zone of the Baltic Sea, a major role in aerosol mercury formation could be caused by Hg (0) reactions with various chlorine species and other halogens (Hedgecock and Pirrone 2001). This may cause the transfer of almost inert, volatile gaseous mercury into reactive gaseous mercury (RGM). High affinity of RGM to water vapor and marine aerosols contributes to the significant increase of mercury deposition (Lindberg and Strattyon 1998). Mercury transformations Hg (0)–RGM–Hg (p) are faster in marine, halogen-rich air (Lin and Pehkonen 1999). The gas-particle conversion also greatly depends on the temperature. During indoor measurements, the temperature was not measured; however, it can be assumed that during heating period, the average temperature in apartments was between 17 and 23 °C. It was similar to the prevailing temperature during mercury measurements in aerosols in non-heating season (18 °C) (Beldowska et al. 2012). Accordingly, one can conclude that the temperature was probably an important factor that influenced the comparable deposition of mercury in house dust during the heating season with outdoor mercury deposition in not-heating period. In order to confirm the above theory, more studies, which would cover parameters such as humidity and air temperature, are needed.

The median of carbon deposition in household dust (70 mg m^−2^ month^−1^) was one and a half times smaller than he deposition of this compound with PM2.5 aerosols in outdoor air (105 mg m^2^ month^−1^) obtained in Gdynia in 2012 during the heating period and exactly one and a half times greater (47 mg m^−2^ month^−1^) than during the non-heating period (Witkowska et al. 2016a). Cao et al. (2005) showed that the proportion of OC in PM2.5 measured in households are changeable depending on their location and is highest in close proximity to streets (from 22.2 to 37.8%), in urbanized areas (20.1 to 38.8%), and in agricultural areas (from 22.1 to 38.9%). At the same time, these proportions are several percent higher in the home environment than outside (56.7 and 43.8 μg m^−3^, respectively).

When analyzing the influence of the external environment on Hg and OC deposition in our households dust, both the location of the building (urban/rural) and its type (flat or a house) were taken into account. Another parameter considered during analysis was the age of the building. In this case, a rough classification was used, which considered all buildings built prior to the year 2000 to be “old” and all those finished from 2000 onwards to be “new.” It was dictated by economic changes that occurred in Poland in the last 20 years and the buoyant growth of the construction industry. Other factors taken into account were the floor on which the measurements were taken, the location of the building in relation to the nearest street (expressed in meters), and the level of traffic. The latter was considered “low” if the number of cars passing per hour was less than 20.

A majority of the dust samples were collected in the Tri-city area (Gdansk, Gdynia, Sopot; Fig. 1), with only 3% in country homes. Both the analyzed dust components, mercury and organic carbon, showed higher depositions in buildings located in the countryside. A small amount of data from rural areas (*N* = 2) made it impossible to conduct statistical tests. However, mercury deposition in buildings located in the countryside was found to be two times higher than in urban locations (228 and 106 ng m^−2^ month^−1^, respectively). This same tendency was noted in the case of organic carbon (101 and 68 mg m^−2^ month^−1^, respectively for rural and urban houses). This is undoubtedly related to the type of fuel used for heating the buildings as individual domestic heating units prevail in the countryside, and heating is obtained by burning coal or biomass, as well as heating fuels of lower quality, and even rubbish (low-capacity DHU) (http://gios.gov.pl). All the dust samples were collected during the heating season (February, March). In the southern Baltic Sea region, this is a time when in the outdoor atmosphere, both in cities and in the country, the depositions of organic carbon and mercury tend to be high, as mentioned above (Lewandowska et al. 2010; Bełdowska et al. 2012). The example of high OC deposition was found in dust collected, in the countryside, in only one of the homes analyzed, that is heated by a coal furnace (126 mg m^−2^ month^−1^). This was accompanied by low Hg deposition amounting to 16 ng m^−2^ month^−1^. On the other hand, the highest mercury deposition occurred in a city home with central heating (1336 ng m^−2^ month^−1^), although it was an outlying value. The level of Hg deposition in that case could have been determined by proximity to a busy street (30 m) and where the traffic level was high. The inhabitants of the flat cooked regularly, smoked much more than 10 cigarettes per day and kept plants and a dog. The OC obtained from the same home was also slightly higher (89 mg m^−2^ month^−1^). The role of all factors indicated above will be shown in detail in the next section; however, our observations suggest that carbon and mercury in indoor dust do not come from the same emission source and their depositions are dependent on several different factors.

**Fig. 3:**
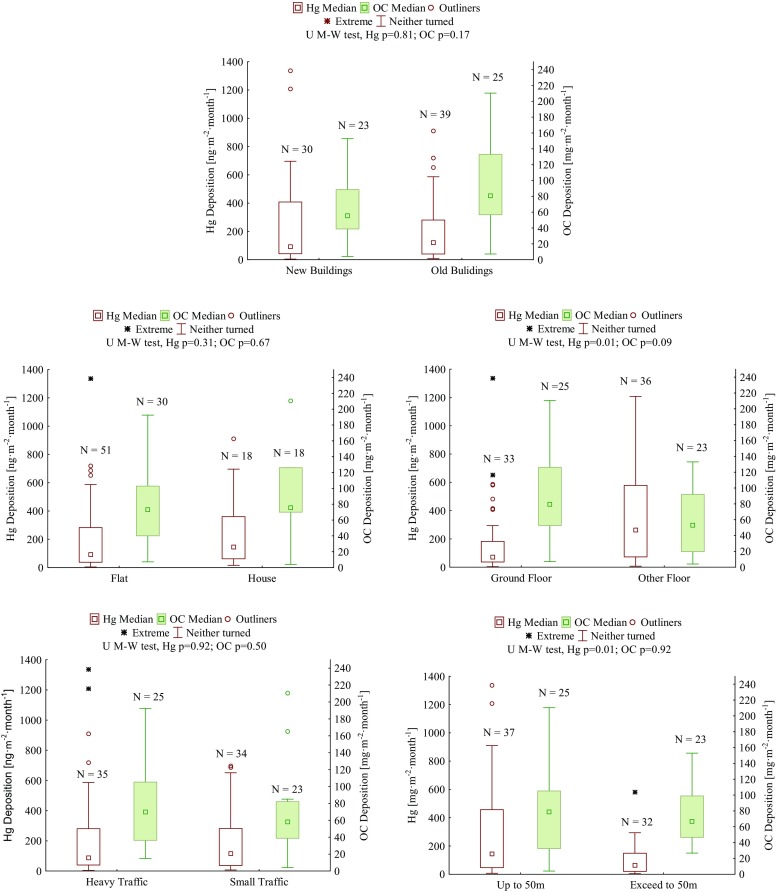
The external factors influencing the concentration levels of mercury and organic carbon deposited in dust. **a** Age of the building. **b** Type of the building. **c** Level of flat/house. **d** Level of traffic. **e** The location of the building in relation to the nearest street [m]

In our research on household dust, we expected much higher depositions of both mercury and organic carbon in the older buildings. This is as a result of the use of latex paint on indoor walls in the late 1980s. Some of the basic ingredients in latex paint were volatile organic compounds which stabilized the layer, and until the 90s, some paints also contained mercury, which has a high accumulative ability (Carpi and Chen 2001). Moreover, until recently, mercury thermometers were still being used in households and it was only the European Council Directive No. 76/769/EWG of 3rd April 2009 that introduced a ban on their use within the EU. Mercury was released into the atmosphere of enclosed spaces if the thermometer was smashed, and at present, the same is true for fluorescent light bulbs. It is currently not possible to indicate statistically significant differences in the depositions of mercury and organic carbon depending on the age of the building where the measurements were taken (Mann-Whitney U test; *p* > 0.05) (Fig. 3a). The median of OC deposition in the dust from old buildings (81 mg m^−2^ month^−1^) was just slightly higher than obtained for new buildings (56 mg m^−2^ month^−1^) (Fig. 3a). Mercury deposition in older buildings was only insignificantly higher than in flats or houses built after the year 2000 (121 and 92 ng m^−2^ month^−1^, respectively). It would appear that the age of a building does not have an essential influence on the shaping of the depositions of the compounds analyzed in this study.

The studied samples collected in the Tri-city agglomeration were also categorized in terms of the type of building the dust came from (Fig. 3b). In the home environment, two types of building were distinguished: (a) a house (a detached, single-family building) and (b) a flat (a multi-family building). In the work environment, samples were collected in buildings that accommodated a restaurant, a pub, a carpentry workshop, a petrol station, a health clinic, an optical laboratory, and a no-longer-in-use laboratory belonging to the Oceanography and Geography Department of the Gdansk University. In the home environment, the highest depositions of both Hg and OC were observed in detached buildings (Fig. 3b) and this is closely related to the use of individual heating units. In the work environment, the highest OC (58 mg m^−2^ month^−1^) was found at the health clinic. The highest Hg deposition was measured at restaurant, visited by many people and with intense cooking (1127 ng m^−2^ month^−1^).

Apart from the factors mentioned above, the analysis of the variability of Hg and OC deposition values in dust also took into account the floor on which the samples were collected and the proximity of the flat or building to the street as well as level of traffic (Fig. 3c, d). In the Tri-city agglomeration, the burning of fuels for transportation purposes is, together with the communal sector, an important source of carbon and mercury in the atmosphere (Lewandowska et al. 2010; Lewandowska and Falkowska 2013). In 2015, the combined number of cars registered in the Tri-city was 288,796. That constitutes about 400 cars per 1000 inhabitants (http://infoekopomorskie.pl), a figure only marginally smaller than in Germany or Finland (530 and 560, respectively), which have the highest number of cars per 1000 inhabitants in Europe (World Bank 2013). Studies on dust conducted in the Tri-city agglomeration helped to determine that the depositions of mercury and organic carbon were at their highest when the house/flat was located no further than 50 m away from the street (Mann-Whitney U test; *p* < 0,05) (Fig. 3e). In the cases where homes were located close to streets with a high level of traffic, OC and Hg were as much as two to three times higher (207 and 213 ng m^−2^ month^−1^, for OC and Hg, respectively) than in the case of streets with smaller traffic (71 and 113 ng m^−2^ month^−1^, for OC and Hg, respectively). An increase in mercury concentration with a rise in traffic intensity in the air in Brno (Czech Republic) was shown by Coufalik et al. (2014). This confirms the results we have obtained for the household dust, considering that deposition to a large extent is determined by the concentration of components in the air.

The mercury deposition measured in the household dust of the Tri-city agglomeration was strongly determined by the floor on which the measurements were taken (Fig. 3c). The mercury deposition median was more than three times higher in dust collected on the ground floor than on higher floors, as confirmed by the Mann-Whitney U test (*p* < 0.05). In the case of organic carbon, the tendency was the reverse of that for mercury. The drop in carbon deposition on lower floors could have resulted from the fact that measurements were taken in winter, when the emission of pollutants from transportation was limited to ground level owing to the height of the mixing layer and thermal inversion. Street configuration could also have had an effect (Tao et al. 2007; Jung et al. 2011). Moreover, there is a possibility that on the higher floor OC aerosols become secondary organic aerosols.

## The influence of internal factors on the deposition levels of mercury and carbon deposited in dust

Morawska and Salthammer (2003) observed that in ventilated rooms, where there is no observable influence of internal factors, the impact of pollutants from external sources can be amplified. However, when there are internal emission sources of given compounds in the room, their role are all the more pronounced. On the basis of questionnaire surveys, it was possible to analyze the influence of factors related to living conditions (all samples included places inhabited by 2 or more people), but also places of work, some of which were visited by up to 150 people a day. Other factors taken into account were type of heating and the presence of plants or pets.

**Fig. 4:**
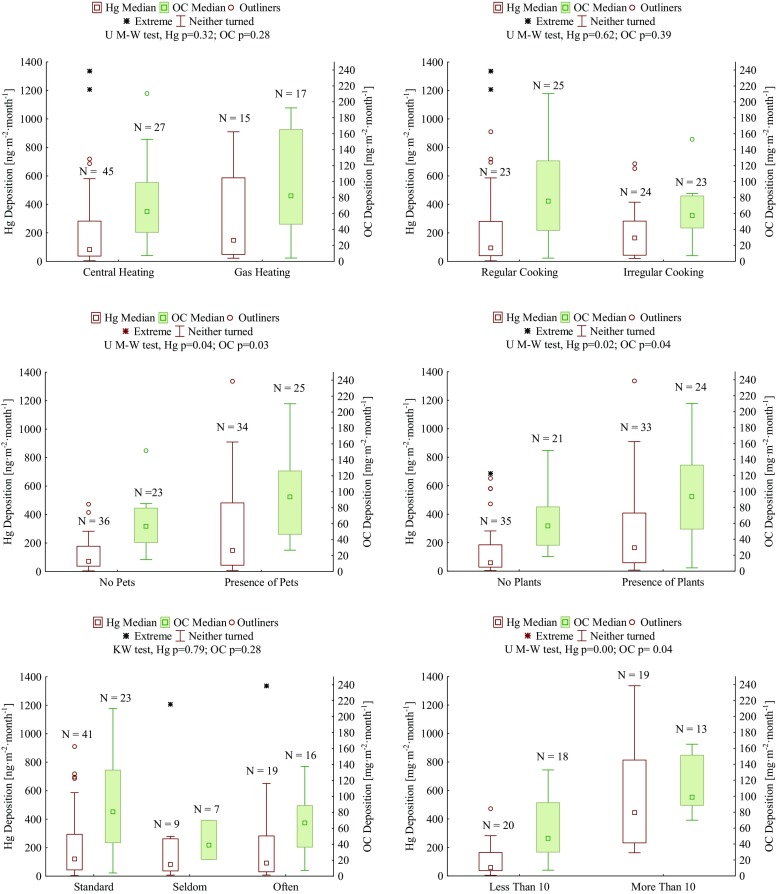
The internal factors influencing the concentration levels of mercury and carbon deposited in dust. **a** Type of heating. **b** Cooking. **c** Presence of plants. **d** Presence of pets. **e** cleaning frequency. **f** Amount of cigarettes

In most EU countries, electricity or gas are used to heat buildings or cook food. However, there is still a large proportion of homes, particularly in Central and Eastern Europe, where solid fuels or biomass are used for heating purposes (Semple et al. 2012; Witkowska et al. 2016a and b). In Poland, more than 88% of produced energy comes from coal energy plants, of which nearly 53% use black coal and 35% brown coal. Providing homes with heat and electricity has the greatest share in Poland’s energy balance. More than 31% of energy is used by households (http://www.stat.gov.pl). The influence of the communal-utility sector on the quality of the surrounding air and variability of carbon and mercury depositions is the focus of much scientific research conducted in the Tri-city agglomeration (Lewandowska et al. 2010; Bełdowska et al. 2012; Lewandowska and Falkowska 2013; Witkowska et al. 2016a and b). In the present paper, we focused on the role of particular heating systems in the shaping of organic carbon and mercury deposition in household dust (Fig. 4a). The studies included homes with central heating (48 households), electric heating (2 households), gas heating (17 households), fireplace heating (1), and a coal stove (1). One of the highest organic carbon deposition values was found in a house located in the countryside, where for heating purposes the coal stove was used (126 mg m^−2^ month^−1^). It seems to be natural, since in winter period, up to 50–70% of the organic carbon can be emitted into the outdoor atmosphere as a result of the combustion of coal for heating purposes (Puxbaum et al. 2007). Additionally, in Poland, the coal quality is not always of the highest standard. The predominant role of coal and biomass burning in increasing carbon concentration in the household dust was indicated also by Schweizer et al. (2007). In turn, Semple et al. (2012) obtained the highest PM2.5 depositions in homes, where wood and peat were used as fuel, followed by households in which black coal was used. In the Tri-city agglomeration, median of OC deposition in dust collected in homes with central and gas heating (62 and 82 mg m^−2^ month^−1^, respectively) was lower than those from households using coal as fuel (126 mg m^−2^ month^−1^). Moreover, higher amplitude of organic carbon deposition was found in dust collected in gas-heated households (Fig. 4a). The deposition of mercury in dust from households with central heating was the smallest one (83 mg m^−2^ month^−1^). Higher deposition of mercury occurred in houses with gas heating (149 mg m^−2^ month^−1^). In Poland, natural gas is used for the heating of households. Sometimes, during technological processing, mercury vapors and metallic mercury is not completely removed from the gas. We suggest that it could be the reason for elevated mercury deposition in dust where gas heating systems were used, as opposed to central heating systems. On the contrary, a coal stove, which was used in only one of the analyzed households, did not translate into a high mercury deposition (16 mg m^−2^ month^−1^). The highest mercury deposition was recorded in a house which utilized a fireplace for heating (1408 mg m^−2^ month^−1^). Because it was only one case, we cannot indicate whether this is a consistent trend.

The frequency of cooking also had an effect on the organic carbon deposition level in household dust (Fig. 4b). Such a dependency was not determined for mercury. Despite the fact that the differences were not statistically significant (*p* > 0.05), it was possible to determine that in flats with regular cooking practices, the median of OC depositions was more than 20% higher than in other homes in which cooking was sporadic or not occurring at all (Fig. 4b). Moreover, in case of regular cooking, the amplitude of deposition was higher. This means that, in the air of enclosed spaces, cooking can be responsible to a greater extent for the emission of OC, as confirmed in earlier studies by Cao et al. (2005). In turn, Buoanno et al. (2014) conducted studies on 24 Italian couples, where the men worked full time and the women were housewives. The authors established that the individual exposition to air pollution with ultra-small particles was greater for women than for men. This was the consequence of activities performed during cooking. High pollutant concentrations can remain in the air, indoors, for long periods of time after the cooking process. (Buoanno et al. 2014).

The variability of the depositions of OC in indoor dust in the Tri-city agglomeration region was also influenced by the presence of plants or pets in the household (Fig. 4c, d). In places where plants were present, the median of OC deposition was almost 40% higher than in homes without plants (94 and 54 mg m^−2^ month^−1^, respectively). That was confirmed by a Mann-Whitney U test (*p* < 0.05). In a home environment, as a result of gas and particle conversion, volatile organic compounds (VOCs), condensation, and physical and chemical adsorption, secondary organic carbon can be formed (Witkowska et al. 2016a) and deposited in dust. The additional sources of OC in household dust could be spores and pollen emitted by plants, as well as particles of soil. Similar observations of higher depositions in the presence of plants were made for mercury. The median of Hg deposition was considerably higher in houses containing plants (164 ng m^−2^ month^−1^) than in those without plants (59 ng m^−2^ month^−1^) (Mann-Whitney U test; *p* < 0.05).

In studies conducted in the Tri-city agglomeration, the influence of pets on the level of OC deposition in home dust was also noted (Fig. 4c). In homes with pets (mainly dogs or cats), despite a higher amplitude of depositions, the OC median was almost 40% higher than in households without the presence of pets. An even greater difference (64%) was observed for mercury. Both are confirmed by a Mann-Whitney U test (*p* < 0.05). The influence of pets could have resulted from the fact that they are carriers of dust and transport pollutants from the external environment. Animals are a good basis for the accumulation of pollutants, as they do not wash often, and harmful substances such as mercury can accumulate in their fur. The deposition of mercury in dog fur can reach the value of 826 ng g^−1^ (Morawska et al. 2013). On the other hand, Semple et al. (2012) showed that the presence of pets can lead to an increase in endotoxins and CO_2_ in the atmosphere. Animals detoxify through shedding, and the shed fur, accumulating on floors and furniture, then becomes a component of household dust. The same goes for plants, which are hardly ever dusted during a standard cleaning session (once a week). Taking the above into account, one of the factors analyzed in the present study was the frequency of cleaning (Fig. 4e). In the Tri-city agglomeration, it was observed that Hg and OC deposition in household dust had a tendency to increase slightly when homes were vacuumed once a week (standard frequency). The results were also characterized by higher amplitude than with other cleaning frequencies. The lowest deposition values of the analyzed compounds were observed when cleaning was performed once every 2 weeks or less frequently. None of the obtained dependencies were statistically significant (Kruskal-Wallis test; *p* > 0.05) and therefore can be treated only as a speculation, that frequent cleaning leads to the resuspension of dust and an increase in the deposition of both analyzed substances. Another reason could be the infrequent changing of filters in vacuum cleaners. In such cases, the dirt collected on filters during earlier cleaning sessions becomes blown out again when the vacuum is being used. The differences between mercury depositions in household dust could have been caused by the use of various detergents that possibly contained mercury (Carpi and Chen 2001).

The number of cigarettes smoked proved to be a very significant factor influencing the level of carbon and mercury depositions in household dust (Fig. 4f). The group of respondents included 33 smokers. It can be assumed that the quality of air will be worse in flats where cigarettes are smoked (Sample et al. 2012). With each cigarette, 8100 μg PM2.5 is emitted into the air and OC can be found in 95% of the mass of these aerosols (Na and Cocker 2005). Smoking cigarettes was indicated as a dominant source of high aerosol depositions in the indoor atmosphere by Hussein and coauthors (2006). In their opinion, smoking one cigarette indoors is equivalent to the amount of aerosols produced during half an hour of cooking, and particles emitted with cigarette smoke can stay in the atmosphere for up to 10 h (Hussein et al. 2006). Cigarette smoke has also been classed as a dangerous pollutant in indoor air, characterized by high potential for exposure (Meng et al. 2005). Studies conducted in the Tri-city agglomeration showed that in flats where cigarettes were regularly smoked, there were higher Hg and OC deposition medians, as opposed to houses with people who are non-smokers (Fig. 4f), although the difference was not statistically significant (*p* > 0.05). The greatest rise in mercury deposition was observed in flats where 10 or more cigarettes were smoked daily. In this case, the dependency was statistically significant (Mann-Whitney U test; *p* < 0.05), and the median of mercury deposition reached 445 ng m^−2^ month^−1^. Organic carbon deposition was also higher and equal to 99 mg m^−2^ month^−1^. Semple et al. (2012) found a similar directly proportional dependency between PM2.5 deposition in indoor air and the number of cigarettes smoked inside. This tendency was most pronounced in homes inhabited by older people who spent a considerable amount of time in enclosed spaces (Semple et al. 2012). Comparing data from a hundred households that used various types of heating fuels, Semple et al. (2012) obtained PM2.5 values that were an order of magnitude higher in the homes of smokers than of non-smokers.

## Summary

Studies into mercury (Hg) and organic carbon (OC) deposited in household dust were conducted in home and work environments between 2013 and 2015, always during winter (heating period). Organic carbon depositions were similar in both the environments, while mercury was characterized by higher deposition in work dust than in dust collected in the house environment.

The median of mercury deposition in household dust was three times lower than outdoor deposition (for fine plus coarse aerosols) obtained by other researchers in the Baltic Sea region during the heating period. At the same time, deposition of this compound in house dust was very close to that obtained outside during the non-heating period. The median of organic carbon deposition in households during the heating period was one and a half times lower than in outdoor air (PM2.5). On the other hand, during the non-heating period, OC deposition in house dust was one and a half times higher than outside.

Among the most important external factors determining the deposition levels of mercury and organic carbon in dust was the location of the building. Both components exhibited higher depositions in places located in the countryside than in the city. In rural areas where individual domestic heating units prevail, heating is obtained by burning coal and biomass as well as heating fuels of low quality. Very often rubbish is also used. However, these results need to be confirmed in further studies, since our data concerned only two samples from rural locations. In a home environment, the highest depositions of both mercury and organic carbon were obtained in dust collected in single-family houses. The depositions of both components were insignificantly higher in older buildings. The fuel which was linked to increased air pollution with organic carbon was black coal, while for mercury, it was natural gas. It is most likely that, during technological processing, mercury vapors and metallic mercury were not completely removed from gas, which resulted in elevated Hg deposition in household dust.

Burning fuels for transportation purposes translated to a rise in the depositions of mercury and organic carbon in home dust. The highest depositions of both compounds were observed in households located in direct proximity to busy streets. Mercury deposition was greater on the ground floor than on higher floors of a building, while for carbon, the reverse is true.

The presence of plants and pets was one of the internal factors shaping the deposition levels of mercury and organic carbon and we found that the frequency of cooking had more of an impact on the rise in OC depositions than Hg depositions within household dust. The levels of mercury and organic carbon depositions were also influenced by smoking cigarettes indoors, with the highest rise in depositions observed when the number of cigarettes smoked inside was greater than 10 per day.
